# Comprehensive analysis on the regulation of differentially expressed of mRNA and ncRNA in different ovarian stages of ark shell *Scapharca broughtonii*

**DOI:** 10.1186/s12864-023-09648-z

**Published:** 2023-09-22

**Authors:** Wenjing Wang, Biao Wu, Zhihong Liu, Xiujun Sun, Liqing Zhou, Wandong Xu, Tao Yu, Yanxin Zheng, Shihao Zhang

**Affiliations:** 1 State Key Laboratory of Mariculture Biobreeding and Sustainable Goods, Yellow Sea Fisheries Research Institute, Chinese Academy of Fishery Sciences, 266071 Qingdao, China; 2 National Demonstration Center for Experimental Fisheries Science Education, Shanghai Ocean University, 201306 Shanghai, China; 3 Laboratory for Marine Fisheries Science and Food Production Processes, Laoshan Laboratory, 266237 Qingdao, China; 4 Administrative Examination and Approval Service Bureau of Kenli District, Dongying, China 257500; 5 Changdao Enhancement and Experiment Station, Chinese Academy of Fishery Sciences, Yantai, China 265800; 6 Shandong Anhai lnvestment , Jinan, China Co., Ltd, 250013

**Keywords:** Ovarian development, Non-coding RNA, Sex steroid hormones, CeRNA, *Scapharca broughtonii*

## Abstract

**Background:**

Ovarian development is an important prerequisite and basis for animal reproduction. In many vertebrates, it is regulated by multiple genes and influenced by sex steroid hormones and environmental factors. However, relative information is limited in shellfish. To explore the biological functions and molecular mechanisms of mRNA and non-coding RNA that regulate ovarian development in *Scapharca broughtonii*, we performed whole transcriptome sequencing analysis on ovaries at three developmental stages. Furthermore, the biological processes involved in the differential expression of mRNA and ncRNA were analyzed.

**Results:**

A total of 11,342 mRNAs, 6897 lncRNAs, 135 circRNAs, and 275 miRNAs were differentially expressed. By mapping the differentially expressed RNAs from the three developmental stages of Venn diagram, multiple groups of shared mRNAs and lncRNAs were found to be associated with ovarian development, with some mRNA and ncRNA functions associated with steroid hormone. In addition, we constructed and visualized the lncRNA/circRNA-miRNA-mRNA network based on ceRNA targeting relationships.

**Conclusions:**

These findings may facilitate our further understanding the mRNA and ncRNAs roles in the regulation of shellfish reproduction.

**Supplementary Information:**

The online version contains supplementary material available at 10.1186/s12864-023-09648-z.

## Background

Gonadal development is an important part of the individual organism development and is the basis for reproductive activity. The stable and sustainable development of shellfish aquaculture industry depends on the supply of high quality and sufficient seedlings [1]. To maintain the steady development of seedling production, an in-depth study on the ovarian development of shellfish is necessary. Ovarian development is regulated by combination of endogenous and exogenous factors, where the endogenous factors are genetic materials such as genes, while the exogenous factors are environmental factors such as temperature, pH and photoperiod, and usually these exogenous factors regulate gonadal development by affecting the expression of the relevant genes [2]. Steroids generally play important roles as hormones in a variety of physiological processes, such as biological sex differentiation, reproduction, development and maintenance of endostasis [3]. Sex steroid hormones play various important physiological roles in vertebrates, including the regulation of growth, development, reproduction, differentiation, metabolism and homeostasis [4]. They mainly include progesterone, testosterone and estradiol, which have traditionally been defined by their role in normal reproductive function [5]. Sex steroid hormones are synthesized under the catalysis action of regulatory proteins and hormone synthase, and the genes encoding these proteins or enzymes mainly include *StAR* (*Steroidogenic acute regulatory protein*), *CYP* (*Cytochrome P450*) and *HSD* (*Hydroxysteroid dehydrogenases*) gene family members. Many earlier studies suggested sex steroid hormones existed only in vertebrates, however, more and more evidences proved they were also found in lots of mollusk [6–9], and varied seasonally with the reproductive cycle [10–12]. Therefore, the sex steroid hormones are also believed to be important roles in shellfish reproductive regulation.

Non-coding RNAs (ncRNAs) are a class of RNAs that do not normally encode proteins but functionally regulate protein expression [13]. Based on the size and biogenesis pathways, they are subdivided into several families including long non-coding RNAs (lncRNAs, with a length of > 200 bp), circular RNAs (circRNAs, with a closed continuous loop), and microRNAs (miRNAs, with a length of about 22 nucleotides) [14]. There are increasing evidences that lncRNAs are potential regulatory molecules as functional roles in high-order chromosomal dynamics, telomere biology and subcellular structural organization [15–17]. Evidences have been accumulating that circRNAs have great potential to exert effects a variety of physiological processes, such as affect microRNA regulation, regulating parental gene transcription, cell proliferation, and RNA-binding proteins [18–20]. Competitive endogenous RNA (ceRNA) regulation hypothesis suggests that mRNA, lncRNA, circRNA and pseudogene transcripts regulate the stability or translation activity of target genes by competitive binding with miRNA, thus achieving post-transcriptional regulation of genes [21–23]. Tang et al. used whole transcript sequencing to discover a dual ceRNA molecular pathway and to resolve the epigenetic mechanism of ceRNA regulation of sex determination and differentiation in Chinese tongue sole (*Cynoglossus semilaevis*), demonstrating for the first time in fish the regulation of sexual development by ncRNA mediated ceRNA crosstalk and revealing a new regulatory mechanism of sex determination and differentiation [24]. However, the studies of ncRNA regulation on shellfish reproduction, especially for the sex steroid hormones, still remain very limited.

The ark shell, *Scapharca broughtonii*, which is widely distributed along the northwest Pacific coast, is a kind of bivalve with important economic value [25]. In recent years, it has become an aquaculture variety with general approval in north coast of China because of its good market prospects, delicious taste and high nutritional value [26]. Because the gonads of *S. broughtonii* are covered by foot muscles, their gonad development stage cannot be judged by naked eyes, which brings trouble to the artificial breeding of seedlings. Therefore, it is very important to study the regularity and regulation of gonadal development. However, little is known about the regulating mechanisms of ncRNA in reproduction by sex steroid hormones of the ark shell. In this study, we performed whole transcriptome analysis for the ovaries from three development periods. The ceRNA networks were mapped based on the correlation between ncRNAs associated with ovarian development. These findings may facilitate our further understanding of ncRNA roles related to ovarian development in the reproductive regulation of the ark shell and provide new information for future genetic breeding in aquaculture.

## Results

### Determination of the period of ovary development

**Fig. 1:**
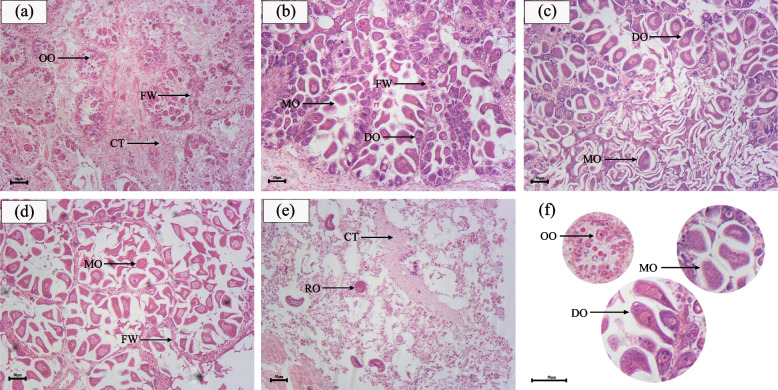
The five stages histological sections of *S. broughtonii* ovaries. Note: **a** Early active stage; **b** Development stage; **c** Ripe stage; **d** Spawning stages; **e** Spent stage; **f** the higher magnification of oogonia, developing oocyte and mature oocyte. CT: connective tissue; FW: follicle wall; OO: oogonia; DO: developing oocyte; MO: mature oocyte; RO: residual oocyte; bar: 50 µm

The gonad development in marine shellfish is distinctly cyclical and can be divided into different periods with the season change. According to previous study [27], the ovary development stage of *S. broughtonii* was divided into five phases based on the observation of ovary sections containing stage I (Early active stage), stage II (Development stage), stage III (Ripe stage), stage IV (Spawning stage) and stage V (Spent stage). The histological sections at five stages were obtained (Fig. 1), and the ovarian development regularity of *S. broughtonii* was similar to most other shellfish species. The oogonia were located on the follicle wall of ovary, and the developing oocytes were connected to the follicle wall of ovary by short stem-like structures, and the mature oocytes wander in the follicle with irregular shape (Fig. 1f). In stage I, follicles were sparse and small in number, and oogonia on follicle wall increased continuously. The number of follicles increased in stage II, and most developing oocytes were connected to the follicle wall by short stem-like structures. Stage III follicles were filled with mature free mature oocytes. The number of mature oocytes in stage IV decreased. Stage V follicles rupture and disperse, leaving only a small number of oocytes, which are gradually absorbed by surrounding cells.

### Sequencing and mapping to the reference genome

After filtration, 172.84 Gb clean reads were obtained from RNA libraries of the nine *S. broughtonii* samples and the Q30 base percentage of each sample was more than 92.06%. Approximately 56.18% ~ 68.89% of all reads were mapped to the *S. broughtonii* reference genome, while 42.64% ~ 54.72% and 8.82% ~ 22.16% were uniquely and multiply mapped, respectively. Additionally, 39.55% ~ 54.80% and 39.61% ~ 54.69 of all reads were mapped to sense strand and antisense strand, respectively. A total of 103.32 M clean reads was obtained after sequencing of the small RNA libraries, with each sample clean data not less than 10.01 M. In total, 45,453 mRNAs, 69,768 lncRNAs, 3810 circRNAs and 1239 miRNAs were detected, and 21,408 novel genes were discovered in this study.

### Expressional differences of mRNAs, lncRNAs, circRNAs and miRNAs

**Fig. 2:**
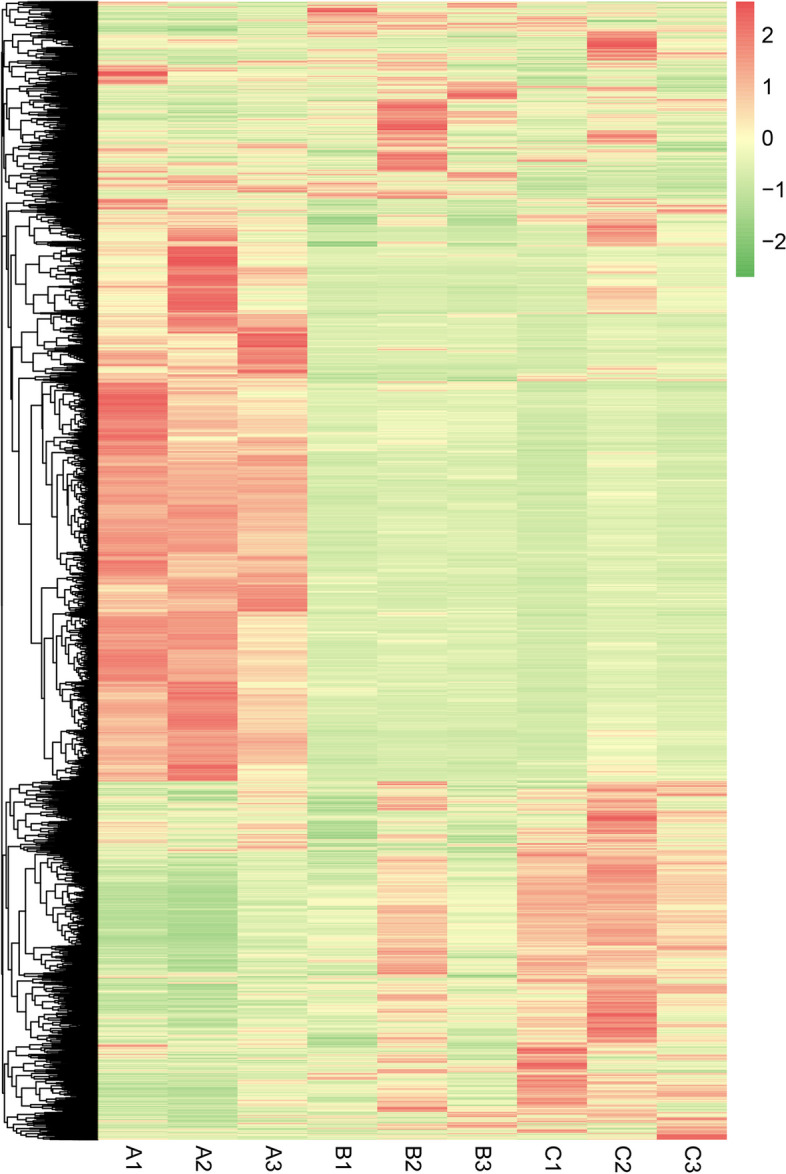
Heat map for DEGs of nine samples

To further explore the global transcript changes, the differentially expressed mRNAs (DEGs), lncRNAs (DELs), circRNAs (DECs) and miRNAs (DEMis) were detected. Compared to group A, a total of 8302 DEGs (4344 up-regulated and 3958 down-regulated), 3788 DELs (1564 up-regulated and 2224 down-regulated), 66 DECs (24 up-regulated and 42 down-regulated) and 164 DEMis (79 up-regulated and 85 down-regulated) was identified in group B, and 8680 DEGs (4599 up-regulated and 4081 down-regulated), 4090 DELs (1866 up-regulated and 2224 down-regulated), 76 DECs (43 up-regulated and 33 down-regulated) and 178 DEMis (76 up-regulated and 102 down-regulated) were identified in group C, respectively. Compared to group B, 1918 DEGs (926 up-regulated and 992 down-regulated), 1622 DELs (903 up-regulated and 719 down-regulated), 38 DECs (14 up-regulated and 24 down-regulated) and 46 DEMis (20 up-regulated and 26 down-regulated) were screened in group C. Additionally, the heat map of the hierarchical clustering analysis clustered genes with the same or similar expression patterns. The hierarchical clustering heat maps of DEGs, DELs, DECs and DEMis expression are shown in detail in Fig. 2 and Fig. S1. The three samples from each group were consistently clustered together in the heatmap, indicating that all DEGs, DELs, DECs and DEMis were highly reproducible. These results demonstrated the high reproducibility and reliability of the transcriptome analysis in this study.

### Functional enrichment analysis of DEGs

**Fig. 3:**
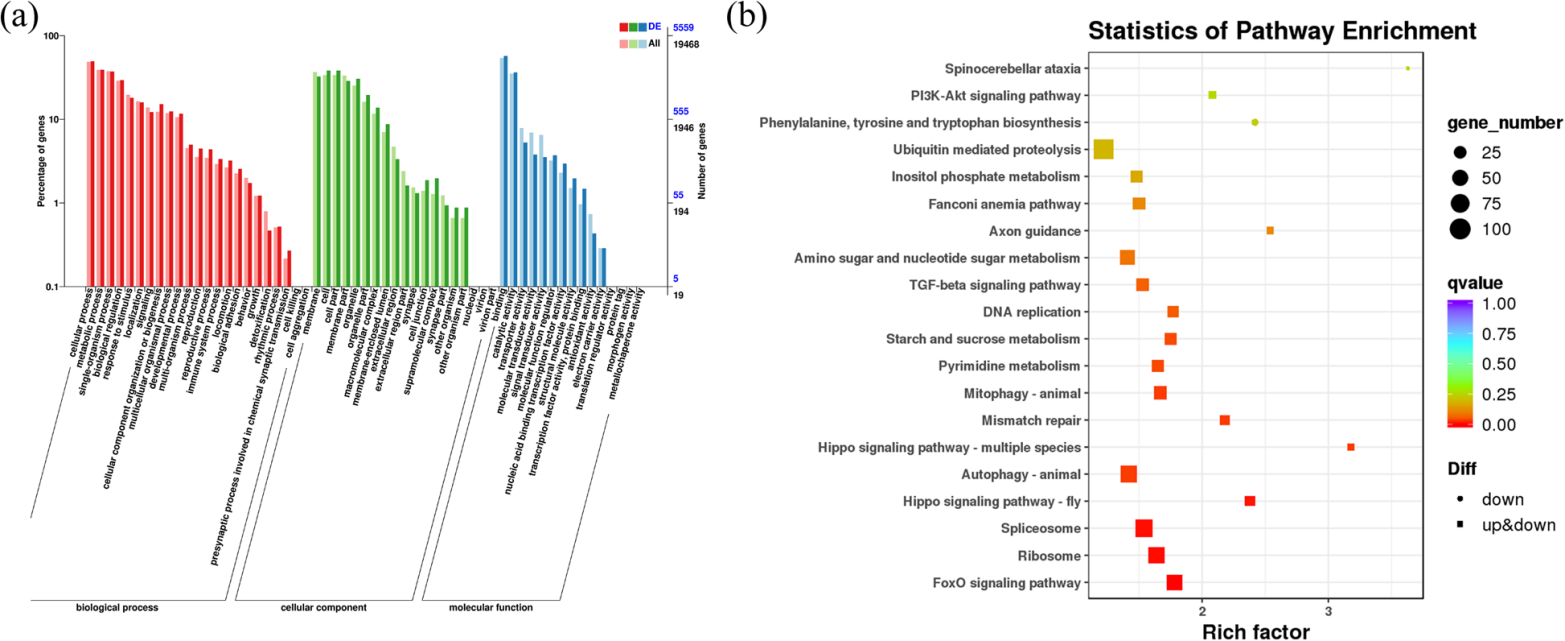
The GO and KEGG enrichment analysis of the DEGs between group A vs B. **a** GO enrichment analysis of DEGs in group A vs B. **b** KEGG enrichment analysis of DEGs in group A vs B

In this study, a total of 11,342 DEGs were identified, and the GO and KEGG enrichment analysis based on those DEGs were performed. It was found that 1243, 1055 and 315 GO terms were significantly enriched (*P*-value < 0.05) in the paired comparison of group A vs B, A vs C and B vs C, respectively. A part of significantly enriched terms for the Biological Process (BP), Cellular Components (CC) and Molecular Function (MF) were displayed in Fig. 3 and Fig. S2. In the GO enrichment analysis, the most significantly enriched GO terms in the three groups was binding and cellular process.

KEGG enrichment analysis was performed on all DEGs to further determine the metabolic process pathways. The results revealed that the DEGs were significantly enriched in 53 KEGG pathways. As shown in Fig. 3 and Fig. S2, the top 20 significant KEGG pathways for each group were chosen and displayed. Of those, the most significantly enriched pathway for DEGs in groups A vs B, A vs C and B vs C were “FoxO signaling pathway”, “Ribosome” and “Oxidative phosphorylation”, respectively. Twenty of the KEGG pathways significantly enriched in the A vs B and A vs C groups were identical, including “Ribosome”, “FoxO signaling pathway”, “Hippo signaling pathway” and “DNA replication”. The “Ribosomal” pathway facilitates oocyte development and may be associated with ovarian development. The primary biochemical and signal transduction pathways identified by the KEGG analysis will provide further insight into the future research direction of mRNA.

**Fig. 4:**
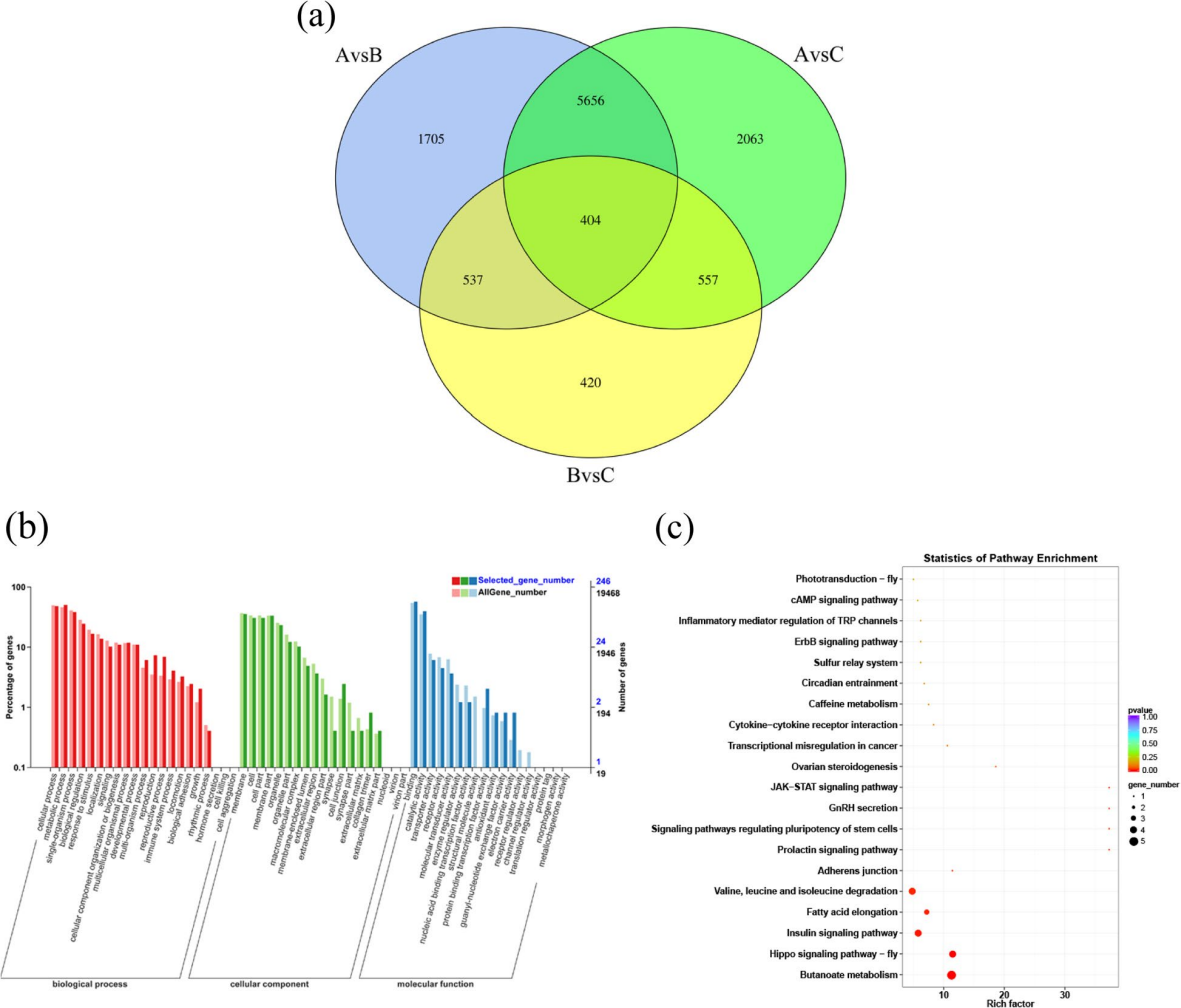
Venn diagram of DEGs among three groups and GO and KEGG enrichment analysis plots of the 404 DEGs shared among groups. **a** Venn diagram of all DEGs among three groups; **b** GO enrichment analysis of the 404 DEGs shared among three groups; **c** KEGG enrichment analysis of the 404 DEGs shared among three groups

To investigate the potential functions of DEGs related to ovarian development, all DEGs from three groups were plotted on a Venn diagram. As illustrated in Fig. 4a, the DEGs shared by the three groups had 404 genes. Of these, some genes were annotated with functions related to gonad development and sex steroid hormones, such as *CYP17A*, *StAR-related lipid transfer protein 10* and *steroid dehydrogenase*. The GO enrichment analysis of those DEGs was shown in Fig. 4b, and the top 10 significantly over-represented GO terms were related to “binding”, “cellular process” and “metabolic process”. As shown in Fig. 4c, the top 20 significant KEGG terms included “Ovarian steroidogenesis”, “GnRH secretion” and “Prolactin signaling pathway” which were involved in ovarian development and sex steroid hormone synthesis.

### Functional analysis of DELs and their target genes

**Fig. 5:**
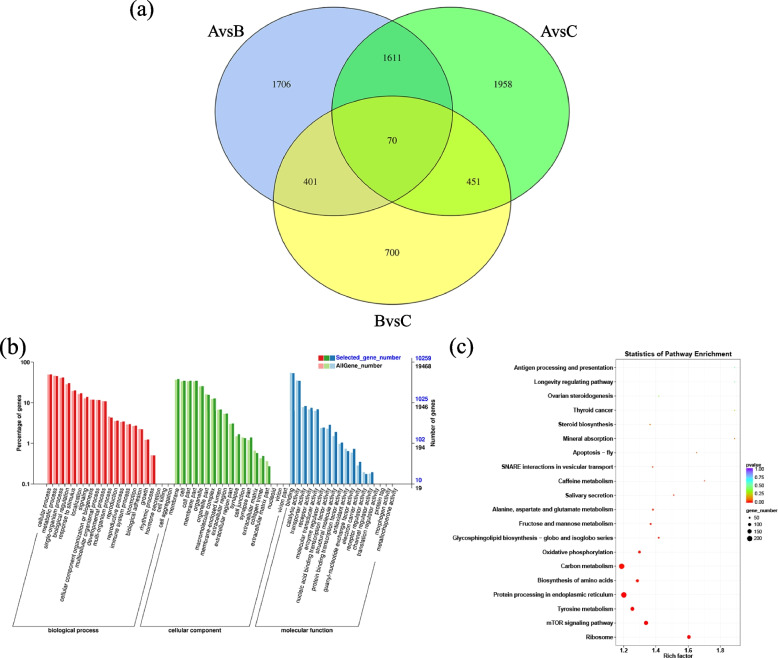
Venn diagram of DELs shared among three groups and GO and KEGG enrichment analysis plots of the target genes. **a** Venn diagram of all DEGs among three groups; **b** GO enrichment analysis on the target genes of 70 DELs shared among three groups; **c** KEGG enrichment analysis on the target genes of 70 DELs shared among three groups

lncRNAs with coding potential were filtered out using the software CPC2, CNCI, CPAT and Pfamscan, and a total of 69,768 lncRNAs were identified. Most of these were defined as intergenic lncRNA, accounting for 75.1%, and followed by intron-lncRNA accounting for 20.9%. Further, using the screening criteria of Fold Change > 1.5 and *P*-value < 0.05, 6897 DELs was obtained. To investigate the possible functions of lncRNA associated with ovarian development, a Venn plot of the three groups was drawn as Fig. 5a. It showed that the DELs shared among the three groups had 70 lncRNAs related to 21,682 target genes. The GO enrichment analysis showed that the top 10 significantly enriched GO terms were “binding”, “cellular process” and “metabolic process” (Fig. 5b). Seven KEGG pathways were significantly enriched (*P* < 0.05), and the top 20 significant KEGG terms included “Steroid biosynthesis” and “Ovarian steroidogenesis” (Fig. 5c). Of these, some target genes were associat with sex steroid hormones, such as *3 beta-hydroxysteroid dehydrogenase*, *17β-HSD14*, *StAR -related lipid transfer protein 5*, *steroid 17-alpha-hydroxylase/17, 20 lyase-like isoform X2*, *Estrogen receptor* and *Steroid receptor RNA activator 1*.

### Functional analysis on DECs

**Fig. 6:**
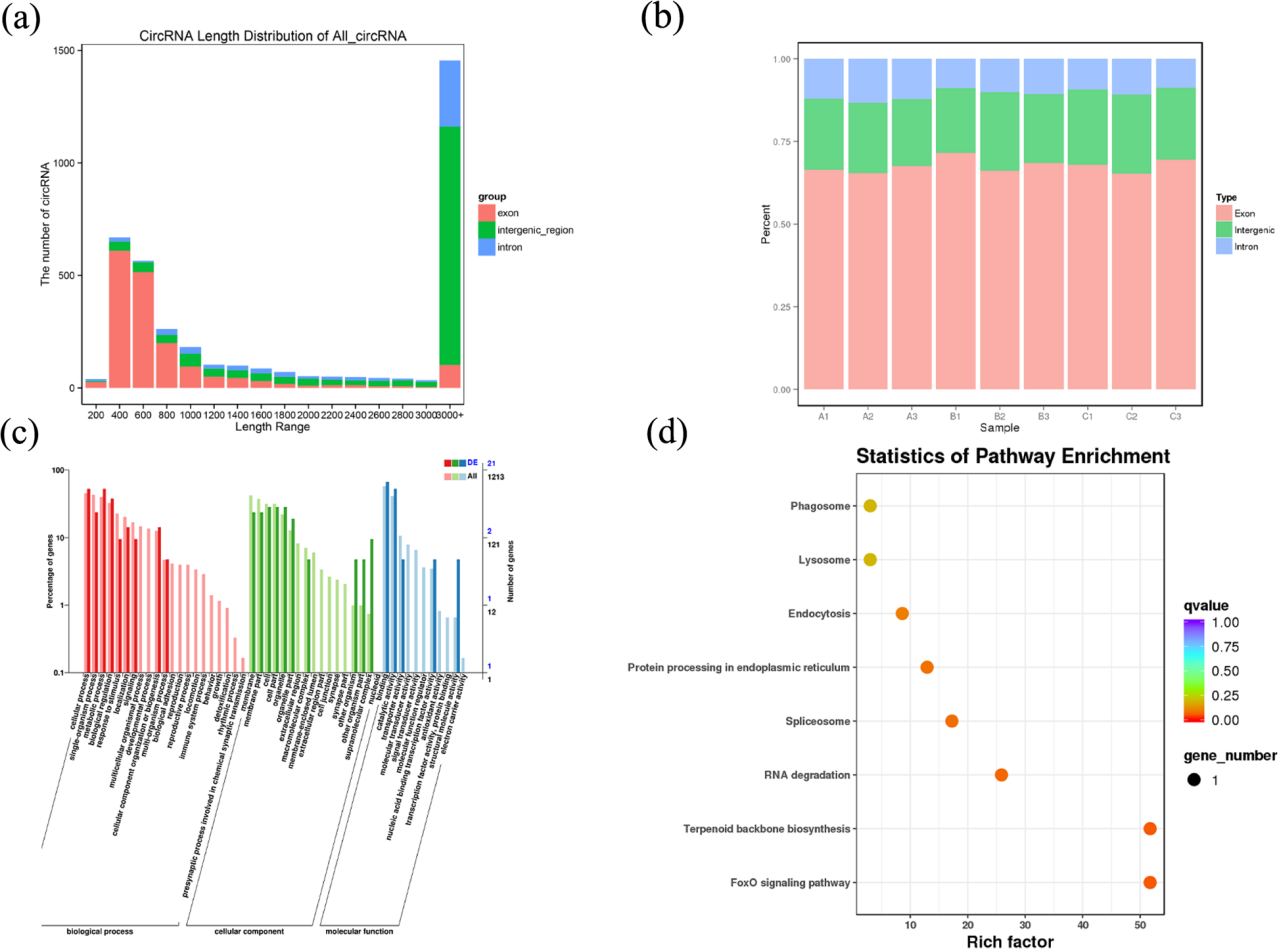
Distribution of circRNAs and GO and KEGG enrichment analysis on DECs host gene in group A vs B. **a** Distribution diagram of circRNA length; **b** circRNA distribution in different regions of the genome; **c** GO enrichment analysis on the DECs host gene in group A vs B; **d** KEGG enrichment analysis on the DECs host gene in group A vs B

The length distribution of circRNAs were analyzed (Fig. 6a), and it was found that the sequence length of most circRNAs were greater than 3000 bp, and followed by the length range from 400 bp to 600 bp. There were 3810 circRNAs were identified using the CIRI software, of which 70%, 20% and 10% belonged to exon, intergenic region and intronic type, respectively (Fig. 6b). And a total of 135 DECs were detected among the three groups by using Fold Change > 1.5 and *P*-value < 0.05 as the screening criteria. We identified 66 DECs in group A vs B, 76 in groups A vs C and 38 in group B vs C. All host genes of DECs were functionally annotated, and the GO enrichment were shown in Fig. 6 and Fig. S3. The binding and cellular processes were the most significant among the three groups. KEGG pathway enrichment analysis was performed on DECs host genes, and 12 KEGG pathways were enriched including “Insulin signaling pathway”, “FoxO signaling pathway” and “Lysosome”.

### Construction of potential lncRNA/circRNA-miRNA-mRNA regulatory networks

**Fig. 7:**
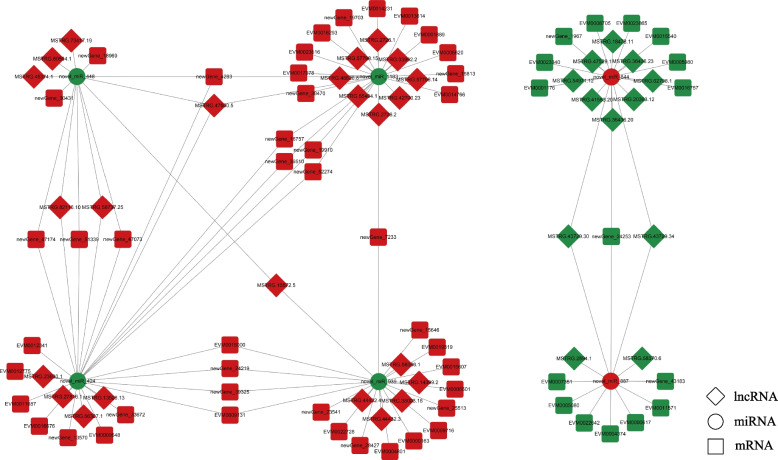
Interaction diagram of lncRNA-miRNA-mRNA network associated with ovarian development. Red and green nodes represent up-regulated and down-regulated RNAs, respectively

**Fig. 8:**
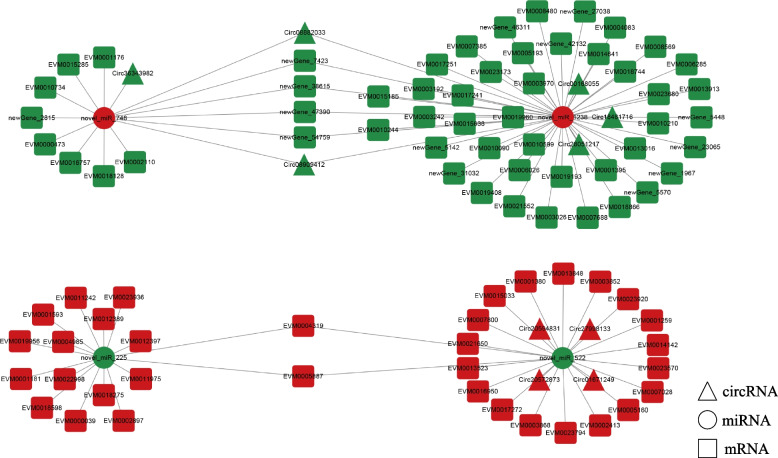
Interaction diagram of circRNA-miRNA-mRNA network associated with ovarian development. Red and green nodes represent up-regulated and down-regulated RNAs, respectively

To identify ceRNA interactions associated with ovarian development, we constructed ceRNA networks based on the interaction of differentially expressed mRNA and ncRNA, and the ceRNA networks were constructed and visualized using the cytoscape software. Among them, part of the network related to ovarian development was shown in Figs. 7 and 8. As shown in Fig. 7, the lncRNA-miRNA-mRNA network related to ovarian development consisted of 6 miRNAs, 36 lncRNAs and 60 genes, among which 4 genes were involved in ovarian related pathways, such as "Oocyte meiosis" and "GnRH signaling pathway". The circRNA-miRNA-mRNA network (Fig. 8) included 10 circRNAs, 4 miRNAs and 87 DEGs, including 6 ovarian development related genes such as *CYP17*, *17β-HSD12* and *β-catenin*. *CYP17* encodes 17α-hydroxylase/17, 20-lyase, a key enzyme in the steroid synthesis pathway with significant roles in steroid synthesis, sex determination and differentiation and gametogenesis. In the ceRNA network, two *CYP17 genes* (EVM0010244, EVM0015185) both interacted with a miRNA (novel_miR_1238), and five circRNAs (Circ00168055, Circ18481716, Circ28051217, Circ08882033, Circ08909412) acted as sponges in this process. *CYP17* is key rate-limiting enzyme in the sex steroid hormone synthesis pathway and plays an important role in steroid hormone synthesis of vertebrates. The novel_miR_1238 interacts with two *CYP17* genes, suggesting that novel_miR_1238 may indirectly regulate *CYP17* genes and thus participate in the regulation of sex steroid hormone synthesis. These results suggest that lncRNA and circRNA may function as ceRNAs, competing with miRNAs as a binding partner of mRNA, which may regulate the metabolism involved in the synthesis of sex steroid hormones.

### Quantitative real-time PCR validation

**Fig. 9:**
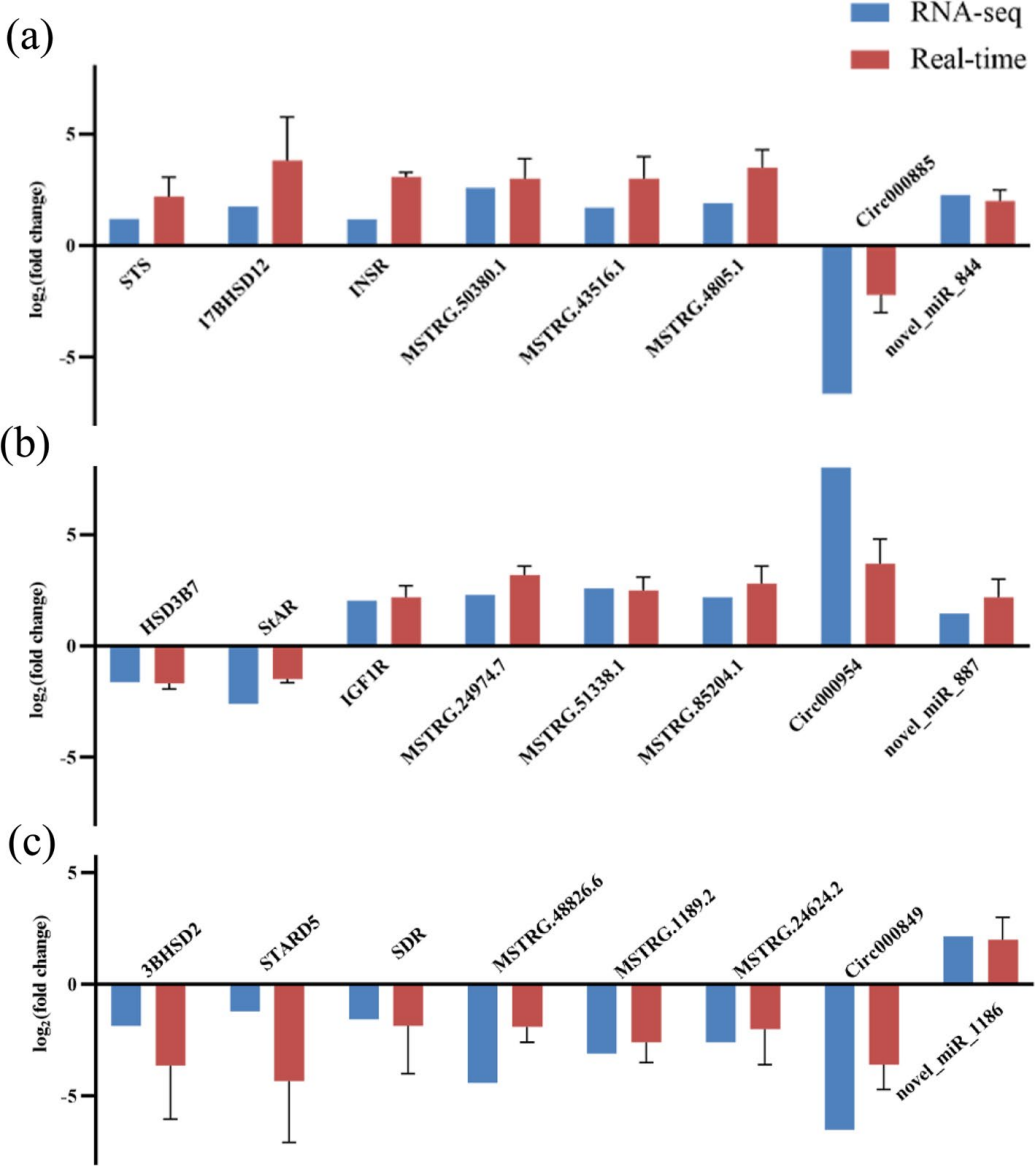
Validation of the RNA-seq data by qRT-PCR. **a** RNA-seq and qRT-PCR data for group A vs B; **b** RNA-seq and qRT-PCR data for group A vs C; **c** RNA-seq and qRT-PCR data for group B vs C

To validate the accuracy and reliability of transcriptome sequence data, a total of 24 differentially expressed RNAs were selected for qRT-PCR analysis, including three mRNAs, three lncRNAs, one circRNA and one miRNA in each groups. As shown in Fig. 9, the qRT-PCR results were coincided with the RNA sequence data, which confirmed the reliability of RNA sequence analysis.

### Expression profile of nine genes related to sex steroid hormone

**Fig. 10:**
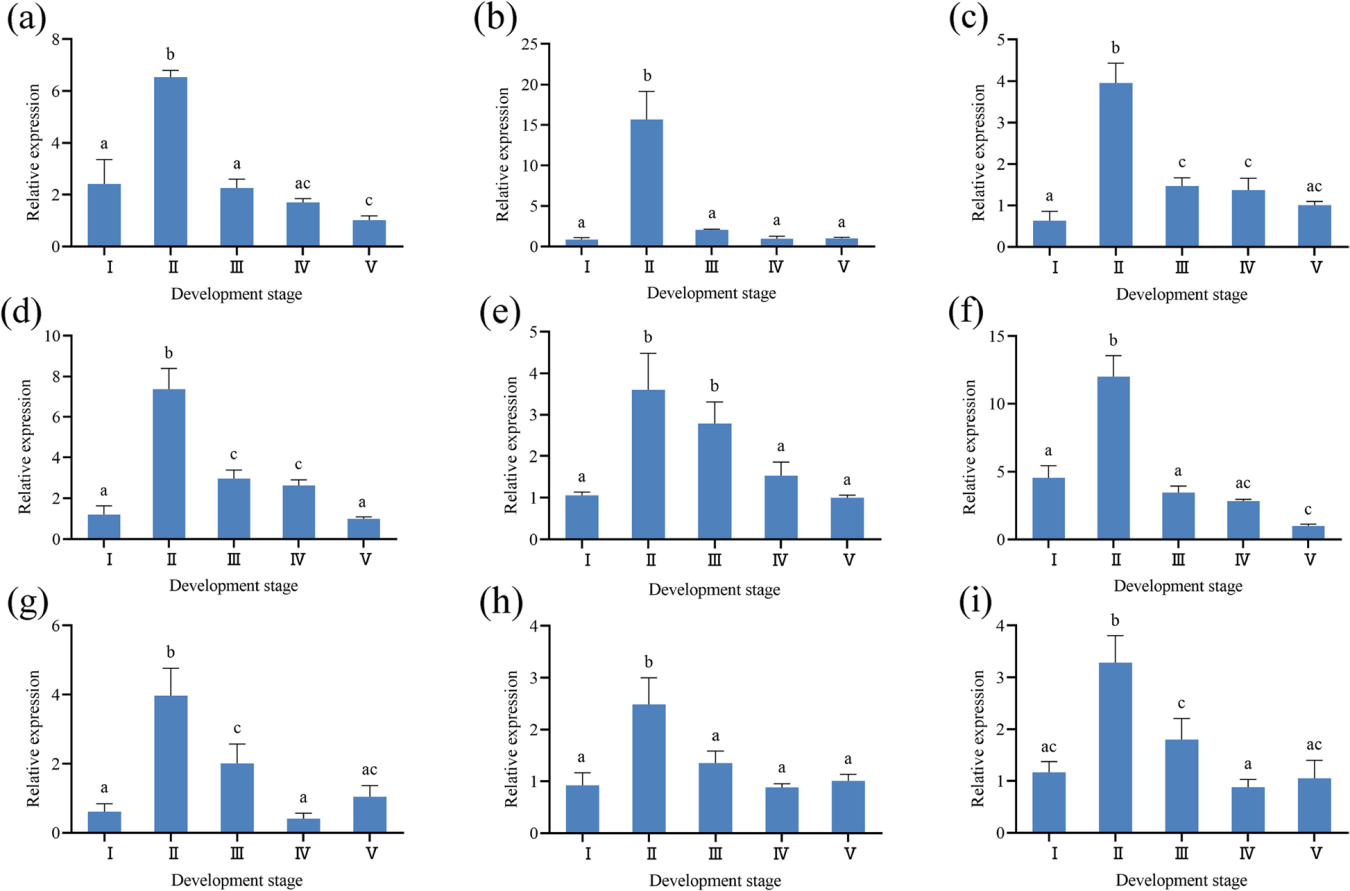
The expression pattern of nine related genes. **a** *17BHSD14*, **b** *CYP17A*, **c** *CYP3A28*, **d** *Steroid receptor*, **e** *START5*, **f** *Beta-catenin*, **g** *3BHSD7*, **h** *CYP17*, **i** *Catenin beta-like*. The different lowercase letters represent significant differences in expression of genes (*P* < 0.05)

The expression pattern of nine genes associated with sex steroid hormones and reproduction in different stages of ovarian development were analyzed. During ovarian development, nine related genes exhibited two different expression patterns. The relative expression levels of *17BHSD14*, *CYP17A*, *CYP3A28*, *Steroid receptor*, *START5* and *Beta-catenin* showed an increasing trend during ovarian development from stage I to stage II, and reached the highest value in stage II, then there was downward trend during ovarian development from stage II to stage V (Fig. 10 a-f). The relative expression levels of *3BHSD7*, *CYP17* and *Catenin beta-like* increased during ovarian development from stage I to stage II, and reached the highest value in stage II, then decreased during ovarian development from stage II to stage IV, and slightly increased during ovarian development from stage IV to stage V (Fig. 10 g-i).

## Discussion

In this study, we explored the different expression patterns of mRNAs and ncRNA for the first time during the different development periods of ovary for screening the key factors influencing the breeding of ark shell at the whole-transcriptome level. In addition, the potential lncRNA/circRNA-miRNA-mRNA regulatory networks were constructed, which provided new insights into the molecular mechanisms underlying the effects of sex steroid hormones on shellfish ovarian development.

DEGs with known functions associated with ovary development and sex steroid hormones synthesis were discovered through comparative transcriptome analysis of overies at different development stages, such as *CYP17A*, *StAR-related lipid transfer protein 10* and *steroid dehydrogenase*. *CYP17A* is a key rate-limiting enzyme in the biosynthesis of sex steroid hormones, progesterone and glucocorticoids [28]. Its activity and expression are important in regulating gonad development, germ cell differentiation and maintenance of secondary sexual characteristics [29]. The expression of *CYP17A* was high in the early stage of gonadal development and has sex dimorphism of *Hyriopsis cumingii*. After interfering with *StAR3* gene, the expression level of *CYP17A* gene in gonad decreases, as did the content of sex steroid hormone, indicating that *CYP17A* gene plays important roles in gonadal development and participates in sex determination and gonadal development through its role in the synthesis pathway of sex steroid hormone [30]. This indicated that these differential expression *CYP17* genes play important roles in the process of ovarian development and sex steroid hormones synthesis. The *StAR* transport of cholesterol from the outer mitochondrial membrane to the inner mitochondrial membrane is the first and rate-limiting step in sex steroid hormone synthesis [31]. Namely, *StAR* is an important gene in the rate-limiting step of the sex steroid hormone synthesis pathway and regulates the rate and level of sex steroid hormone synthesis. In *Chlamys farreri*, *StAR3* gene was highly expressed in the gonadal tissue at the early stage of differentiation, and then decreased again in the ovaries at maturity after its expression decreased during growth, which may be because *StAR3* provides an important enzyme for steroid hormone synthesis in the early stage of ovarian cell differentiation and ovarian maturation [32]. The *StAR-related lipid transfer protein 10* may play an significant role in the transport of cholesterol during the early stages of ovarian development. The expression levels of *CsDMRT3* and *CsFOXL2* genes related to reproduction reached their peak in the mature stage of testes and ovaries development in the clam *Cyclina sinensis*, respectively. *CsDMRT3* and *CsFOXL2* expression levels were highest in the gonads when estradiol was treated with 5 µg/L, and oocyte diameter became larger with the increase of estradiol concentration. The results showed that, *CsDMRT3* and *CsFOXL2* play important roles in gonadal development and sex differentiation of *C. sinensis* [33, 34].

Non-coding RNAs, as important members of the intracellular gene regulatory network, influence various cellular life activities. Current studies suggest that non-coding RNAs also play important roles in the reproductive activities and participates in the regulation of spermatogenesis and oogenesis [35]. In recent years, researchers have discovered and identified a large number of non-coding RNA, but the studies on non-coding RNAs are very limited in aquatic animals. In this study, 69,768 lncRNAs, 3810 circRNAs and 1239 miRNAs were identified in nine samples, and 6897 DELs, 135 DECs and 164 DEMis were also detected. In mammals, ncRNAs have been shown to play a crucial role in the development of gonads [36, 37]. High-throughput sequencing of porcine ovaries revealed that circRNAs may play potential roles in regulating porcine litter size [38]. Currently, the main focus in aquatic animals is on the post-transcriptional regulation of non-coding RNA. Through miRNA sequencing analysis of Chinese mitten crab (*Eriocheir sinensis*) ovary, Song et al. found that miR-2 and miR-133 were differentially expressed during meiotic maturation in oocytes, indicating that miRNA may regulate *cyclin B* expression at the post-transcriptional level [39]. Transcriptome sequencing of the testes and ovaries of large yellow croaker (*Larimichthys crocea*) revealed multiple lncRNA targeting known genes related to sex differentiation and developmental. Among them, lncRNA MSTRG.24346 is extremely significant correlated with a sex determination candidate gene *dmrt1*, indicating that lncRNA may play an important role in sex differentiation [40]. In this study, we analyzed the expression changes of ncRNAs during ovarian development in ark shell, and screened the differentially expressed ncRNAs during different ovarian development stages by comparative analysis, and further performed target gene prediction and biological function annotation of differentially expressed ncRNAs with the help of bioinformatics tools. Functional annotation revealed that some lncRNAs were involved in reproductive signaling pathways such as steroid biosynthesis, ovarian steroidogenesis and estrogen signaling pathway involving target genes such as *3β-HSD*, *17β-HSD14*, *StAR-associated lipid transfer protein 5*, *estrogen recepto*r and *steroid receptor RNA activator 1*, which are related to reproduction.

According to the ceRNA hypothesis, lncRNAs and circRNAs can function as miRNA's molecular sponges to restore the expression of mRNAs that miRNA has suppressed. The primary pathway of ncRNA-mRNA interactions is through ceRNA networks. Functional research of miRNA has found that ceRNA (most commonly lncRNA and circRNA) can regulate gene expression by competitively binding to miRNA. The ceRNA can affect miRNA-induced gene silencing through the miRNA via miRNA response elements (MREs) [41]. Sequencing of the longissimus dorsi muscle from two different pigs identified potential lncRNAs/circRNAs-miRNAs-mRNAs regulatory networks sharing genes involved in skeletal muscle muscular proliferation, differentiation/regeneration, and adipogenesis. These differentially expressed non-coding RNA as ceRNA for miRNAs may be involved in the molecular basis of muscle traits [42]. By mapping the known ovarian development related genes in ark shell, the ceRNA of the differentially expressed non-coding RNA and target genes were also analyzed. The lncRNA-miRNA-mRNA network consisted of 6 miRNAs, 36 lncRNAs and 60 genes, among which 4 genes were involved in ovarian related pathways. The circRNA-miRNA-mRNA network showed that a total of 101 RNAs interacted with each other, including 10 circRNAs, 4 miRNAs and 87 DEGs. And this circRNAs-miRNAs-mRNAs regulatory network includes six genes related to ovarian development. A total of 10 miRNAs associated with ovarian development were identified in this study, all were novel miRNAs. Furthermore, 36 lncRNAs and 10 circRNAs participated in the lncRNA/circRNA-miRNA-mRNA networks related to ovarian development, respectively, which may play potential roles in ovarian development. The lncRNA/circRNA-miRNA-mRNA network analysis revealed that miRNAs target and regulate genes were associated with ovarian development and sex steroid hormones. We found that the novel_miR_1238 regulates two *CYP17* genes, which encode 17α-hydroxylase/17, 20-lyase that are key enzymes in the sex steroid hormone synthesis pathway. *CYP17* gene controls the direction of steroid synthesis and is one of the key rate-limiting enzymes in sex steroid hormones biosynthesis pathway [43]. Therefore, novel_miR_1238 may indirectly regulate *CYP17* gene to participate in sex steroid hormone synthesis.

## Conclusions

We compared the expressional characteristics of mRNAs and ncRNAs in the ovarian tissues of *S. broughtonii*. According to the results, ncRNAs were abundant in the ovarian tissues, and bioinformatics analyses indicated that ncRNAs were involved in ovarian development and sex steroid hormone. We identified 10 miRNAs, 36 lncRNAs and 10 circRNAs that may be involved in the regulation of ovarian development. This study provides new information about the effects of genetic and ncRNA on shellfish ovarian development and sex steroid hormone synthesis and provides new insights into future genetic breeding for aquaculture.

## Methods

### Sample

A total of 200 *S. broughtonii* individuals with an average shell length of 80.74 ± 5.08 mm at different stages of gonadal development were collected from Yantai in Shandong Province. Before sampling, all ark shells were cultured in tanks containing fully aerated filtered seawater at salinity of 30 ± 1, temperature of 18 ± 1 °C, pH of 7.75 ± 0.25 and dissolved oxygen of 7.56 ± 0.23 mg/L for one week. During the culture period, 100% of the seawater was replaced daily with fresh seawater, and fresh single-celled algae was provided every four hours. All sampled gonad tissues were orderly dissected, placed in liquid nitrogen, and then stored in a freezer at –80 ℃. Simultaneous, part of dissected gonads were placed in Bouin's fixative to fix for 24 h and then stored in 70% of the ethanol, used in paraffin sections to determine the gonad development period. Gonadal tissue sections were visualized using a Leica microscope to determine the specific periods of gonad development. In the following studies, nine ovarian samples were randomly selected from stages I (group A), stages III (group B), and stages IV (group C) for subsequent sequencing, with three replicates per group.

### RNA extraction and quality monitoring

Nine ovarian samples from three developmental stages, with three replicates, were selected for RNA extraction. Total RNAs were extracted by using the miRNeasy Mini Kit (Qiagen, Valencia, CA, USA) according to the manufacturer’s instructions. RNA concentration was measured using Nanodrop 2000 (Thermo Fisher Scientific, Wilmington, DE, USA), and RNA integrity was analyzed with agarose gel electrophoresis and the Agient 2100 Bioanalyzer (Agilent Technologies, Foster City, CA, USA). Samples with an RNA integrity number (RIN) value greater than seven were used for further analysis. The nine RNA samples that met the requirements were used for further constructing transcriptome library and sequencing.

### RNA library construction and sequencing

Both the RNA libraries and the small RNA libraries were constructed using 3 µg RNA per sample. After removing ribosomal RNA (rRNA) using the Ribo-ZeroTM kit (Epicentre, Madison, WI, USA), the RNA libraries were built using the VAHTS Universal V6 RNA-seq Library Prep Kit for Illumina® (Vazyme, China) for mRNA, lncRNA and circRNA sequencing. The small RNA libraries were constructed using the VAHTSTM Small RNA Library Prep Kit for Illumina (Vazyme, China) after rRNA was removed. The concentration and insert size were detected using Qubit 3.0 and Agilent LabChip GX 2100, respectively, following the construction of the libraries. The libraries were sequenced on the Illumina NovaSeq 6000 platform (Illumina, San Diego, CA, USA).

### Reads mapping and transcriptome assembly

In order to ensure the accuracy of information analysis, quality control of raw data was required to obtain high-quality reads order, which were clean reads. The clean data was obtained by removing low-quality reads containing over 10% of poly (N) and adapters from the raw data. And then the FastQC (http://www.bioinformatics. babraham.ac.uk/projects/fastqc/) was used to verify the sequence quality. The clean data were mapped to the *S. broughtonii* reference genome [44] sequence by the HISAT2 (version 2.0.4). Mapped reads were assembled into transcripts using StringTie (version 1.3.1). The assembled transcripts were annotated using the gffcompare program (http://ccb.Jhu.edu/software/stringtie/gffcompare.shtml).

### Identification of lncRNA, circRNA, and miRNA

The identification of lncRNA were carried out by two steps including basic screening and potential coding capability screening. The transcripts were selected firstly by exon number ≥ 2, length ≥ 200 bp and the FPKM ≥ 0.1. And then, the coding potential calculator 2 (CPC2) [45], coding-non-coding index (CNCI, v2) [46], Coding Potential Assessment Tool (CPAT) [47] and Pfamscan (version 1.3) [48] were used to filter out the transcripts with coding potential. Transcripts that pass through all those above procedures were considered lncRNAs. CircRNAs were identified with the CIRI software [49]. The CIRI used the BWA software [50] to mapping with the reference gene sequence to generate SAM files. The CIGAR values in the SAM file were then analyzed, and the PCC (Paired Chiastic Clipping) signals was scanned from the SAM file. We mapped the reads to the reference genome to the mature sequences of the known miRNA in the miRBase (v22) database and their upstream and downstream for known miRNAs identification. For the biometric features of miRNAs, the new miRNAs were predicted using miRDeep2 software (version 2.0.5) [51]. The possible precursor sequences were obtained by reads alignment to the location information on the genome. Based on the distribution information of reads on the precursor sequence and the precursor structure energy information, the Bayesian model was scored to finally realize the prediction of the new miRNAs.

### Target gene prediction and annotation

Neighjacent genes within 100 kb upstream and downstream of lncRNA were used as cis target genes using a Perl script. Trans target genes of lncRNA were predicted with correlation coefficient value > 0.9 and significance *P*-value < 0.01. Target gene of miRNA was predicted by using miRanda [52] and targetscan [53] based on the gene sequence information of the known miRNA and the newly predicted miRNA with the corresponding species. Since circRNAs comprise several miRNA binding sites, it is possible to identify circRNAs that bind to miRNAs using miRNA target gene prediction, which may then be used to determine the function of this subset of circRNAs based on the functional annotation of miRNA target genes. The predicted target gene sequences were aligned to the databases of NR, Swiss-Prot, GO, COG, KEGG, KOG and Pfam to obtain the annotation information of the target genes.

### Differentially expressed RNA, GO and KEGG enrichment analyses

The DESeq software package (http://bioconductor.org/packages/release/bioc/html/ DESeq.html) was used to detect differentially expressed genes, differential expressed lncRNAs, differential expressed circRNAs and differential expressed miRNAs by satisfying different value of log2FoldChange|and FDR. The ClusterProfiler software was used to perform Gene Ontology (GO) enrichment and Kyoto Encyclopedia of Genes and Genomes (KEGG) [54] pathway enrichment analysis on DEGs, DELs, DECs and DEMIs, respectively. The KEGG enrichment analysis of differential genes used hyper-geometric distribution testing with ClusterProfiler software. GO terms and KEGG pathways with Benjamin-corrected *P*-value < 0.05 were considered to be significantly enriched.

### Construction of the lncRNA/circRNA-miRNA-mRNA network

It has been proved that the lncRNAs/circRNAs could regulate the mRNA expression by competitively binding to the MREs. To explore the possible interactions between miRNA and mRNA, lncRNA and circRNA, respectively, the miRanda software was used to analyze the combinative ability between the *S. broughtonii* miRNA and the DEMs, DELs and DECs. Based on the ceRNA targeting relationship, the lncRNA/circRNA-miRNA-mRNA network was constructed and visualized using the Cytoscape software V3.9.0 (San Diego, CA, USA). Since lncRNA, circRNA, miRNA and mRNA can form endogenous competitive mechanisms, special attention was paid to target genes negatively correlated with miRNA expression levels in the analysis. That only follow up—down—up "or" down—up—down "trend of the expression of selected for further research.

### Quantitative real-time PCR validation

The accuracy of omics data was validated by the relative expression levels of selected mRNAs, lncRNAs, circRNAs and miRNAs using quantitative real-time PCR (qRT-PCR). In the qRT-PCR experiment, the β-actin [55] was set as internal reference genes for mRNA, lncRNA, and circRNA and mir-125-x [56] for miRNA. The RNA used in the qRT-PCR was the same as that used in sequencing. The cDNA for mRNA, lncRNA and circRNA amplification was converted by HiScript® III RT SuperMix for qPCR Kit, and then the qRT-PCR reactions were performed using the ChamQ SYBR Color qPCR Master Mix Kit according the manufacturer’s instructions. Meanwhile, for miRNA, miRNA 1st Strand cDNA Synthesis Kit and miRNA Universal SYBR qPCR Master Mix were respectively used for cDNA synthesis and qRT-PCR. Each qRT-PCR experiment was carried out in triplicate. Relative gene expression levels were calculated using the 2^−∆∆Ct^ method. The primers designed by Primer 3.0 were listed in Table 1. All data were analyzed using one-way ANOVA in SPSS 22.0. Quantitative data were presented as means ± standard deviation (SD), and the significance level for the statistical analysis was set as *P* < 0.05.

**Table 1:** Primers used in qRT-PCR experiment

Type	Gene name	Sequences (5′-3′)
mRNA	STS	F: TTGGAGGAGAAAGGGATGAATGG R: AGGTACCCTTATCCCTCCATCAA
	17BHSD12	F: GTGTGGTGAAGAGGTTGACTGGAG R:CACGGGTGGACTTTAGGAGAGTTG
	INSR	F: AGCATTCCGTGTAGCCTGTGATTG R: CTGCCGCCATTGATGACCTGTC
	HSD3B7	F: GAGGGTTGTTATGACCAGTTCCT R:CTGTCCAGTCCTCCTCTGTAAAC
	StAR	F: TCCGCCAGCCTCTGTAATAGTCTAC R: TGATCGTTTCGGCATGAGTGACAG
	IGF1R	F: TAACCTCCCTGATCTTGGACATCCC R: GACATGCTGCCTGGCTGGTTTC
	3BHSD2	F: CAGCTCAATCTGGTGGAAAACTG R: GGGATCTGACCTCATCGCTTTAT
	STARD5	F: GGACATGGACCCAGAAACTGTAT R: GTTTGTTAGGTTTCACTGGCTCC
	SDR	F: TTGGAAAGGAAGGGGCTGAAGAATG R: AGGGGCAGACAACACAAACTCAAC
lncRNA	MSTRG.50380.1	F: GAAAGTCACGGTTCTTTCGGATG R: CGCTTATGGCATTATACAGCAGG
	MSTRG.43516.1	F: TTCCACCTGGTATTTCCAAGACC R: GTGCATTAGACCATTCAGCTACAG
	MSTRG.4805.1	F: GGCGCAGACAAATACGGTATTC R: GTCACATGTTAGACACCCTCTGT
	MSTRG.24974.7	F: GGAGACCTTTTTGCCCACATTAG R: GGCAATATTCCCCAATTCTGCAC
	MSTRG.51338.1	F: AGAGCTTCTCTAAGTCAGTGCAC R: CCTAGCATAGACGGACTGAAGAC
	MSTRG.85204.1	F: GGGATCTACCAGTGTACCATGTG R: GACCAAATTTCCCATAGTGGCAC
	MSTRG.48826.6	F: AAGCAGGGTCACGGCAACTAATG R: CGCCCACAATGTTCTGACAAAGC
	MSTRG.1189.2	F: TGGTAGAGTGCTCGCTCCGTAAG R: TTCCCATCCGAAAGAACCGTGAC
	MSTRG.24624.2	F: GTCCACGTCCCACACTTAGAAAGG R: CGACGCCCAGGAAACTTCAGAG
circRNA	Circ000885	F: TGTATGCTGTGTCTGGGACG R: CTCCATCCTGCTGAGTTTAACG
	Circ000954	F: TCACTATCGAGTCACAGTCAAGTCT R: AGGGCTTGTGACTTGTTGTTAGG
	Circ000849	F: CGTTTTACGCCTTCCGGTGT R: TCAACTCTTCCCCTTCATCTCCA
miRNA	novel_miR_844	F: CGCGTAGGTTAGACACGTGAACT R: AGTGCAGGGTCCGAGGTATT
	novel_miR_887	F: TGGGCCTAATGGCTCGG R: AGTGCAGGGTCCGAGGTATT
	novel_miR_1186	F: GCGTTACCCTGTTGAACCGA R: AGTGCAGGGTCCGAGGTATT
Reference gene	β-actin	F: GGTTACACTTTCACCACCACAG R: ACCGGAAGTTTCCATACCTAAGA
	mir-125-x	F: CGCGTCCCTGAGACCATAAC R: AGTGCAGGGTCCGAGGTATT

### Expression changes of nine genes during ovarian development

Three ovarian samples were randomly selected from each orary development stage, and from the identified DEGs, nine genes associated with sex steroid hormones and reproduction were selected for the qRT-PCR experiment, including *CYP17A*, *CYP17*, *CYP3A28*, *3BHSD7*, *17BHSD14*, *START5*, *Steroid receptor*, *Beta-catenin* and *Catenin beta-like*, then their expressionwere analyzed as aboved mentioned. The primers designed by Primer 3.0 were listed in Table 2.

**Table 2:** Primers used in qRT-PCR experiment of related genes

Gene name	Sequences (5′-3′)	Gene name	Sequences (5′-3′)
17BHSD14	F: GGATGGCATGGCAGAAATGT R: CAATACCGCTTGTTCCTCCG	Steroid receptor	F: CTGTGGTCTTTCGGATGCTG R: GATGGAAGCCGAAGAAGCAG
CYP17A	F: TCCACACCGTTTTCTGGACT R: TTCACCAAGACAAACACGGC	START5	F: GGTGAGCCTTATACAGCCAGA R: TCTCCATCATCCTTTAGCGCT
3BHSD7	F: TCCGGTGATGTGGTATTGGT R: CATGGTCCGCTTGTTCTTGT	Beta-catenin	F: TGCAACAACCAACGCAACAA R: TTCTCTGTCTCCAGCCTGGA
CYP3A28	F: GAGTTCCTTCCCTTCCGACA R: TGTTGACCGATACACACCCT	Catenin beta-like	F: GCTGTGTGTGCTTTGAGACA R: ACGGCTTGGTGGATGAAGTA
CYP17	F: AGAGACAAAACCCTCCTCCG R: TCGCCGTATATCTTTGCCCA		

### Supplementary Information

**Additional file 1.**

## Data Availability

All sequencing data was deposited in the NCBI Short Read Archive (SRA) database under the BioProject ID: PRJNA948553. Relevant supporting data can be found within the article and additional files.

## Abbreviations

ncRNAs: non-coding RNAs
lncRNAs: long non-coding RNAs
circRNAs: circular RNAs
miRNAs: microRNAs
ceRNA: competitive endogenous RNA
BP: Biological Process
CC: Cellular Components
MF: Molecular Function
StAR: Steroidogenic Acute Regulatory Protein
MREs: miRNA response elements
RIN: RNA integrity number
rRNA: ribosomal RNA
PCC: Paired Chiastic Clipping
DEGs: differentially expressed genes
DELs: differentially expressed lncRNAs
DECs: differentially expressed circRNAs
DEMis: differentially expressed miRNAs
GO: Gene Ontology
KEGG: Kyoto Encyclopedia of Genes and Genomes
qRT-PCR: quantitative real-time PCR
SD: standard deviation
