# Supplementary material for: Comprehensive analysis on the regulation of differentially expressed of mRNA and ncRNA in different ovarian stages of ark shell Scapharca broughtonii
Source: BMC Genomics. 2023 Sep 22;24:563. doi: 10.1186/s12864-023-09648-z (PMC10515027; doi:10.1186/s12864-023-09648-z)
Supplement: Supplementary file 1 — Additional file 1. [file 12864_2023_9648_MOESM1_ESM.pdf]

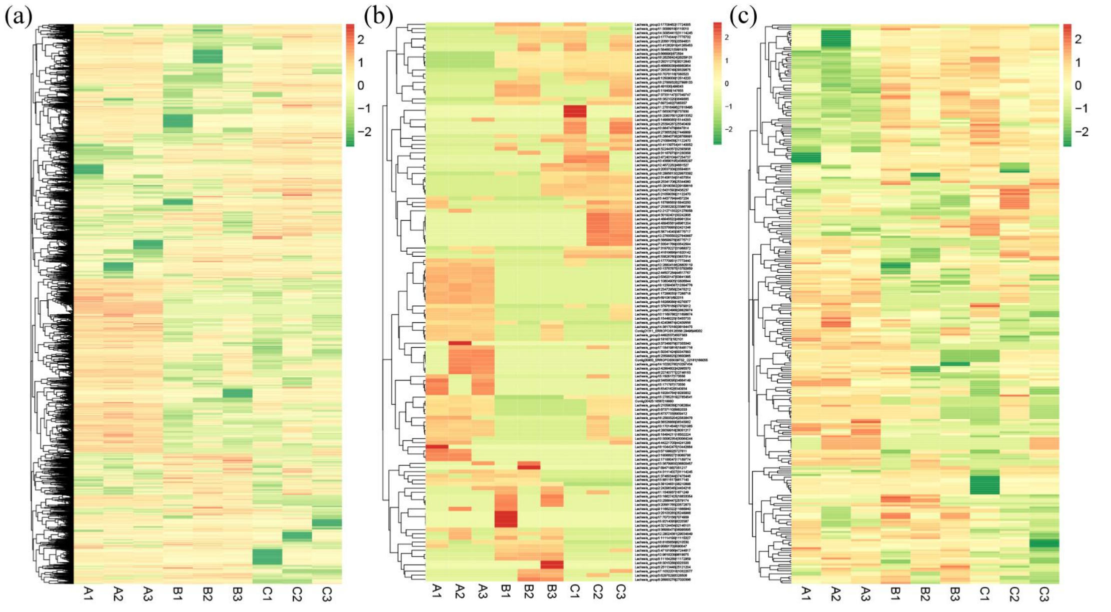

Figure S1. Heat map for DELs (a), DECs (b) and DEMis (c) of nine samples

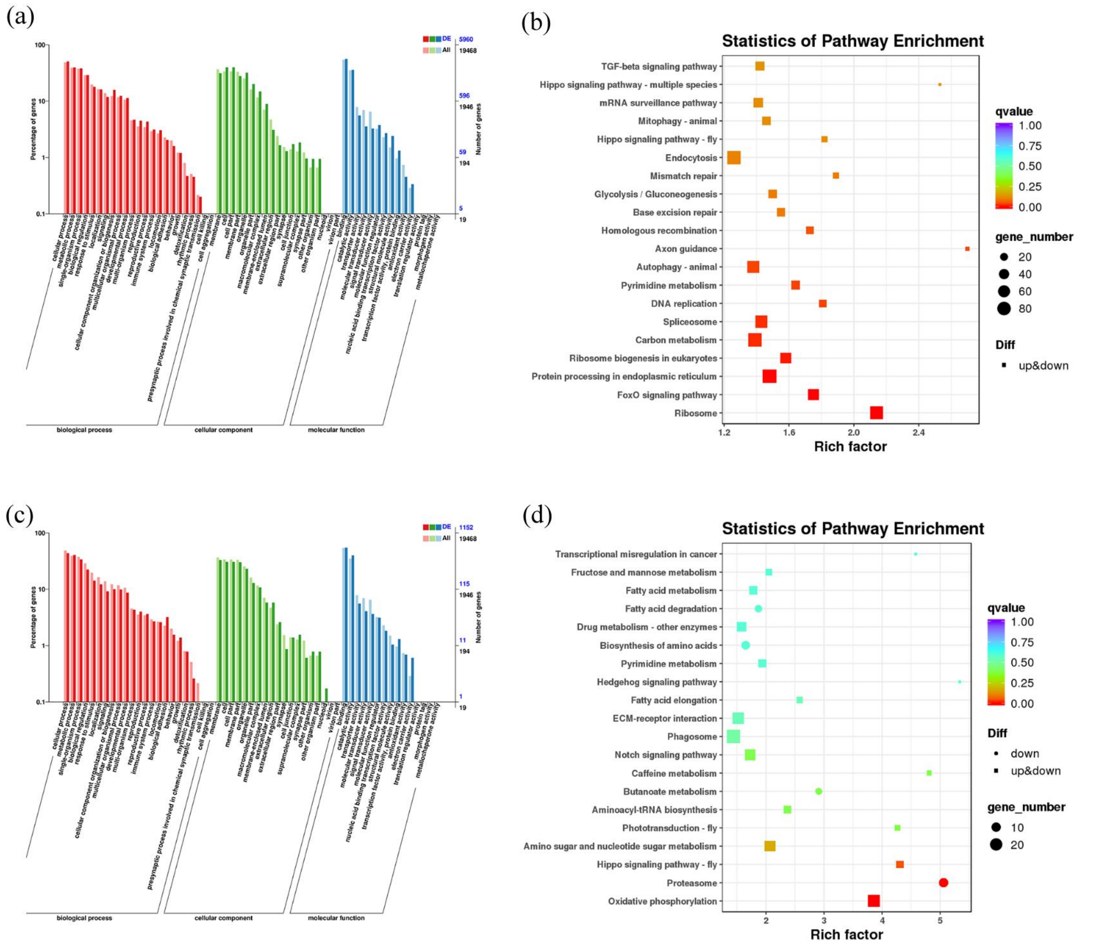

Figure S2 The GO and KEGG enrichment analysis of the DEGs between groups A vs C and Group B vs C. (a) GO enrichment analysis of the DEGs between groups A vs C. (b) KEGG enrichment analysis of the DEGs between groups A vs C. (c) GO enrichment analysis of the DEGs

between groups B vs C. (d) KEGG enrichment analysis of the DEGs between groups B vs C.

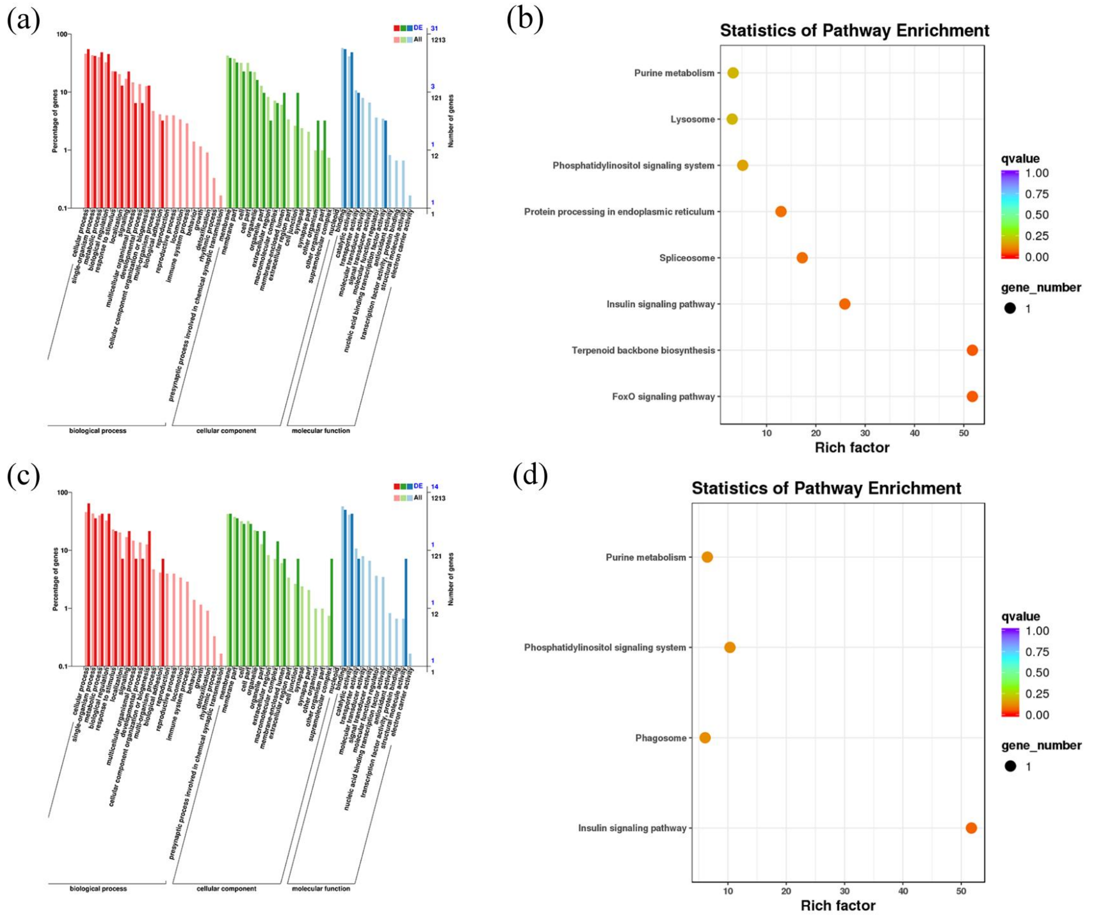

Figure S3 The GO and KEGG enrichment analysis of the DECs' host gene between groups A vs C and Group B vs C. (a) GO enrichment analysis of the DECs' host gene between groups A vs C. (b) KEGG enrichment analysis of the DECs' host gene between groups A vs C. (c) GO enrichment analysis of the DECs' host gene between groups B vs C. (d) KEGG enrichment analysis of the DECs' host gene between groups B vs C.
